# Splice Variants of Perlucin from *Haliotis laevigata* Modulate the Crystallisation of CaCO_3_

**DOI:** 10.1371/journal.pone.0097126

**Published:** 2014-05-13

**Authors:** Tanja Dodenhof, Frank Dietz, Sebastian Franken, Ingo Grunwald, Sørge Kelm

**Affiliations:** 1 Centre for Biomolecular Interactions Bremen, Department of Biology and Chemistry, University Bremen, Bremen, Germany; 2 Department of Biochemistry and Molecular Biology, Rheinische Friedrich-Wilhelms- University, Bonn, Bonn, Germany; 3 Department Adhesives and Chemistry of Polymers, Fraunhofer Institute for Manufacturing Technology and Advanced Materials (IFAM), Bremen, Germany; Centro Nacional de Biotecnologia - CSIC, Spain

## Abstract

Perlucin is one of the proteins of the organic matrix of nacre (mother of pearl) playing an important role in biomineralisation. This nacreous layer can be predominately found in the mollusc lineages and is most intensively studied as a compound of the shell of the marine Australian abalone *Haliotis laevigata.* A more detailed analysis of Perlucin will elucidate some of the still unknown processes in the complex interplay of the organic/inorganic compounds involved in the formation of nacre as a very interesting composite material not only from a life science-based point of view. Within this study we discovered three unknown Perlucin splice variants of the Australian abalone *H. laevigata*. The amplified cDNAs vary from 562 to 815 base pairs and the resulting translation products differ predominantly in the absence or presence of a varying number of a 10 mer peptide C-terminal repeat. The splice variants could further be confirmed by matrix-assisted laser desorption ionisation time of flight mass spectrometry (MALDI-ToF MS) analysis as endogenous Perlucin, purified from decalcified abalone shell. Interestingly, we observed that the different variants expressed as maltose-binding protein (MBP) fusion proteins in *E. coli* showed strong differences in their influence on precipitating CaCO_3_ and that these differences might be due to a splice variant-specific formation of large protein aggregates influenced by the number of the 10 mer peptide repeats. Our results are evidence for a more complex situation with respect to Perlucin functional regulation by demonstrating that Perlucin splice variants modulate the crystallisation of calcium carbonate. The identification of differentially behaving Perlucin variants may open a completely new perspective for the field of nacre biomineralisation.

## Introduction

The mollusc shell consists of two calcium carbonate polymorphs; the outer layer of calcite and the inner iridescent aragonite, or nacre [1]. Nacre itself consists of aragonite tablets arranged in a “stack of coins” [2], [3]. These aragonite tablets are surrounded by the organic matrix, which contains chitin and proteins, like silk fibrinogen-like proteins, secreted by the mantle epithelia [4], [5]. The interplay of the organic matrix and aragonite leads to its structural robustness [6], which makes nacre a composite of interest for material sciences. It is probable that amorphous CaCO_3_ is initially formed as a precursor of crystal growth [7], [8]. If nacre nucleation is induced on glass slides, a sheet of proteins is first secreted onto the substrate, which then is overlaid by a CaCO_3_ calcite sheet. Once this layer is confluent, an abrupt production of aragonite sets in [9]. Belcher et al. [10] showed that regulation, nucleation, growth and aggregation do not need pre-organized organic arrays and that polyanionic proteins are required for aragonite crystal formation. In early shell formation there is a predominately irregular growth of calcite with low expression levels of proteins, followed by more regular growth of calcite on top of the first nacre layer controlled by the organic matrix and mediated by a significant increase of protein expression [11]. Thus far those water-soluble proteins of abalone nacre that have been studied include Perlucin [12]–[14], Perlustrin [13], [15], Perlwapin [16], Perlinhibin [17], Perlbikunin from *H. laevigata* and Lustrin A [18]–[21] AP7, AP8 [22], AP24 [20], [23]–[25] from *H. rufescens*. Further proteins reported to be involved in biomineralisation are Nacrein [26], the N16 family (N16–1, N16–2, N16–3) [27], [28] N66, N14 [29], MPP1 [30] or Pif 97 and Pif 80 [31]. Perlucin from *H. laevigata* was first purified and characterised as a water-soluble protein of 155 amino acids from the nacreous layer [12], [13]. Based on the amino acid sequence a synthetic gene has been designed [GenBank: FB705690.1], although the natural coding sequence has not yet been determined. Perlucin comprises a C-type lectin with a lectin domain spanning the first 130 amino acids followed by two repeats of ten amino acids each [12]. It has one predicted *N*-glycosylation site at position 84. Furthermore, it is able to bind lactose and mannose, most likely via its lectin domain [12]. However, the physiological function of this activity has not yet been investigated.

Together with other proteins of the organic matrix, one function proposed for Perlucin is the nucleation of aragonite crystals [13], [32] by binding to aragonite instead of calcite, [33]. In calcium carbonate precipitation experiments, native Perlucin clearly leads to faster precipitation of CaCO_3_ [13]. Using atomic force microscopy it has been demonstrated that 100 µg/mL Perlucin supports nucleation on calcite in saturated CaCO_3_ solutions [32]. Moreover, if Perlucin is dialysed against a saturated CaCO_3_ solution, the CaCO_3_ crystals formed incorporated Perlucin, which has recently been confirmed by Weber et al. [34] using green fluorescent protein (GFP) tagged Perlucin. Purified native Perlucin always appears very heterogeneous. This observation raised the idea that additional variants exist beside the described protein [12]. Here we describe the cloning and expression of cDNAs encoding Perlucin from the mantle epithelia of *H. laevigata*. Three new splice variants of Perlucin were identified, which differ mainly in the number of a 10 amino acid repeating unit at their C-termini. Recently, it has been assumed that aragonite-associated proteins have evolved signature sequence traits of intrinsic disorder and a predicted “disordered domain” within the repeating region of a Perlucin variant described here has been identified [35]. Interestingly, in recombinant Perlucin the number of repeats strongly influences the precipitation behaviour of CaCO_3,_ suggesting specific physiological roles of the splice variants in the regulation of crystal growth.

## Methods

### RNA Isolation and cDNA Synthesis

A fresh specimen of the Australian abalone *H. laevigata* was obtained as a kind gift from Great Southern Waters (Great Southern Waters Pty. Ltd., The Esp Indented Head, Victoria, Australia). The 10 cm specimen was cooled down on ice for 1 h. Afterwards the mantle epithelium was dissected and immediately frozen in liquid nitrogen. Deep-frozen mantel epithelium was homogenized and total RNA was isolated by using the SV Total RNA Isolation System (Promega, Madison, USA) following the manufacturer’s instructions. Concentration and quality of the RNA were measured using a Nanodrop (Peqlab, Erlangen, Germany). First strand cDNA was performed by using 1 ng of total RNA as a template for RevertAid M-MuLV Reverse Transcriptase (Fermentas, Thermo/Fisher Waltham, MA, USA) and oligo dT primer following the manufacturer’s protocol. Degenerated Primers (sense 5′-TGYTACTGGTTCWSN-3′) and (anti sense 5′-YTTYTGRCAYTGRTARTCRTTCCA-3′) were created based on the amino acid sequence for Perlucin (GenBank: P82596) from the Australian abalone *H. laevigata*. PCR was performed using 1 µL of first strand cDNA and GoTaq polymerase (Promega, Madison, USA) at 50°C annealing temperature. As a control we used primers for actin (sense 5′-GTCACCAACTGGGACGA-3′) and (anti sense 5′-ACCTGACCTCGGGAA-3′) based on the available sequence of *H. tuberculata* actin (GenBank: AM236595.1). PCR products were ligated into pJET1.2 vector (Fermentas), transformed into *E. coli* strain XL1-Blue and clones were sequenced (Max Planck Institute for Marine Microbiology, Bremen, Germany) and edited using the Geneious software [36].

### RACE-PCR

To obtain full-length cDNA of Perlucin we used rapid amplification of cDNA-ends (RACE) as described [37] and followed the manufacturer’s instruction (Roche Applied Science, Mannheim, Germany). For elucidating the 5′ ends we used sequence specific primer (antisense SP1 5′-GTTCAGATCAGA AGCACCAAG-3′) and a second sequence specific primer (antisense SP2 5′-GACCAAGCCAGTAATTGAAAGC 3′). For clarifying the 3′ ends we used a third specific primer (sense SP3 5′-ATATGGCTGTGGGAAGGACAACGCC-3′).

### Plasmids

Plasmids were constructed by standard recombinant cloning techniques and all changes were verified by DNA sequencing. For the amplification of full-length Perlucin splice variants, nested PCR was performed using oligonucleotides: (outer sense 5′-GAGTTGAAGTCACCCGTCATG-3′) and (inner sense 5′-CGGGATCCATGCACGTCGAGGTCCTGTCT-3) and (outer antisense 5′-TGGAAAACAAACATGTTACAG-3′) and (inner antisense 5′-CGGAATTCCAGTTCCTTTTCACAAAT-3′) specific for Perlucin-R0 and (outer antisense 5′-ACTGTCCCTCTGTTACAG-3′) and (inner antisense 5′-CGGAATTCCAGGTTTCCATGCAAACT-3′) specific for Perlucin-R5 and Perlucin-R8. The inner oligonucleotides containing the sequences for *BamHI* and *EcoRI* sites were annealed and cloned into *BamHI*/*EcoRI*-digested pcDNA3 Strep vector [38]. The pcDNA3 Perlucin Strep constructs were also used for transient transfection of COS-7 cells. The coding sequences of the different Perlucin splice variants were amplified out of the pcDNA3 Strep vector. The oligonucleotides (sense 5′-GCCCATGGGATGCCCACTCGGA-3′) and (anti sense Strep-tag specific 5′-GCGCGGCCGCTTATTTTTCGAACTGCGG-3′) containing the sequences coding for *NcoI* and *NotI* sites were annealed and cloned into *NcoI*/*NotI* digested pETM-41 (provided from European Molecular Biology Laboratory (EMBL), Heidelberg, Germany) vector coding for the expression of a N-terminal His-tag followed by MBP. Plasmids were transformed into bacterial expression strain Rosetta (DE3) pLacI (Novagen, Merck KG, Darmstadt, Germany).

To also obtain MBP with a C-terminal Strep-tag we amplified His-MBP using oligonucleotides (sense 5′-GCCCATGGGCCATCACCATCACCATCAC-3′) and (antisense 5′-GCGAATTCGCCCTGAAAATAAAGATTCTC-3′) taking the pETM 41 vector as a template. The oligonucleotides containing the *NcoI* and *EcoRI* sites were annealed and cloned into *NcoI*/*EcoRI* digested pET28b HDGFStrep-tag plasmid [38]. The coding sequence for HDGF had been removed by digesting with *NcoI*/*EcoRI*, while the coding sequence for the Strep-tag remained in the plasmid.

### Phylogenetic Analysis

DNA fragments from *Haliotis spec.* were obtained by searching with BLASTN using Perlucin-R8 cDNA and screening GenBank for Perlucin from *Haliotis spec*. For comparison a range of functional diverse C-type lectins from vertebrates and invertebrates were selected (Table 1). Full length protein sequences were first aligned using T-Coffee server [39] and then truncated at the N-terminus and C-terminus starting from the first cysteine and ending at the last cysteine of the C-type lectin domains, respectively. DNA sequences encoding these proteins fragments were back translated in RevTrans, version 2.0 [40] (http://www.cbs.dtu.dk/services/RevTrans-2.0/web/) and aligned based on the T-Coffee alignment obtained for the amino acid sequences (alignment shown in supporting information S1). Best parameters (HKY85 substitution model [41] with 4 invariant gamma rate categories, HKY85 G+I) for phylogenetic constructions were determined using MEGA5 [42] and applied in the phylogenetic calculations using the MrBayes 3.1 [43] plug-in of Geneious [36], using type II antifreeze glycoprotein from Atlantic herring (*Clupea harengus*) [GenBank: S65819.1] as out-group.

**Table 1:** List of genes used in phylogenetic analysis.

Abbreviation	GenBank	Remarks	*Scientific name*
Venome1	[AF354270]	Venome gland 1	*Bungarus fasciatus*
Venome2	[AF354271]	Venome gland 2	*Bungarus fasciatus*
Venome3	[AF354272]	Venome gland 3	*Bungarus fasciatus*
CLEC1	[NM_072099.5]		*Caenorhabditis elegans*
CLEC48	[NM_075146.1]		*Caenorhabditis elegans*
CLEC49	[NM_075428.3]		*Caenorhabditis elegans*
CLEC50	[NM_075429]		*Caenorhabditis elegans*
antifreeze	[S65819.1]	type II antifreeze glycoprotein	*Clupea harengus*
Hasi499	[GT275204.1]		*Haliotis asinine*
Hasi551	[GT272273.1]		*Haliotis asinine*
Hddperl1	[EF103332]		*Haliotis discus discus*
HddPerl3	[EF103334]		*Haliotis discus discus*
HddPerl4	[EF103335]		*Haliotis discus discus*
HddPerl5	[EF103336]		*Haliotis discus discus*
HddPerl6	[EF103337]		*Haliotis discus discus*
HddPerl7	[EF103338]		*Haliotis discus discus*
HddPerl8	[EF103339]		*Haliotis discus discus*
HdivPerl	[GU446716]		*Haliotis diversicolor*
HdivPerl1	[JN314429]		*Haliotis diversicolor*
HdivPerl4	[JN314433]		*Haliotis diversicolor*
HdivPerl5	[JN314431]		*Haliotis diversicolor*
HdivPerl6	[JN314432]		*Haliotis diversicolor*
HlaevPerl0	[FN674442]		*Haliotis laevigata*
Hmid1a1	[EU135915]		*Haliotis midae*
HvarPerl	This study		*Haliotis varia*
asialo	[NM_001197216.2]	asialoglycoprotein receptor H1	*Homo sapiens*
CD209	[NM_001144899.1]	DC-SIGN	*Homo sapiens*
collectin12	[NM_130386]	collectin sub-family member 12	*Homo sapiens*
DCSIGNR	[AF245219.]	sDC-SIGN1A type III	*Homo sapiens*
LSIGN	[AY343913.1]	CD209L	*Homo sapiens*
macrophlec2	[D50532.1]	macrophage lectin 2	*Homo sapiens*
REG1B	[NM_006507.3]	regenerating islet-derived 1 beta	*Homo sapiens*
Scavrec	[AB038518.1]	scavenger receptor	*Homo sapiens*
versican	[AB209491.1]	chondroitin sulfate proteoglycan 2	*Homo sapiens*

### Transfection of COS-7 Cells

COS-7 (kidney fibroblasts from the African green monkey *Cercopithecus aethiops*) [44] (Leibniz Institute DSMZ, German collection of Microorganisms and cell cultures, Braunschweig, Germany) were grown in DMEM supplemented with 10% foetal bovine serum, 100 U mL^−1^ penicillin and 50 µg mL^−1^ streptomycin at 37°C in a humidified atmosphere of 5% (vol/vol) CO_2_. For transient transfection, cells were grown to a density of 80% confluence. Directly before transfection, the media was changed from 10% foetal bovine serum to 2% foetal bovine serum. Transfection was carried out using polyethylenimine (Sigma-Aldrich, St. Louis, MO, USA) as described [38]. For protein expression, COS-7 cells were transfected with the indicated amount of pcDNA3 Perlucin plasmids and the supernatant were harvested after 24 h. Cell culture supernatants were acetone precipitated using 4 times sample volume and incubated overnight at −20°C. Proteins were precipitated by centrifugation at 20000×g at 4°C for 20 minutes, dissolved in SDS-PAGE sample buffer and loaded on a 12% polyacrylamide gel.

### Protein Expression in *E. coli*

Overnight cultures of transformed *E. coli* Rosetta (DE3) pLacI were used to inoculate 1000 mL of LB media (1∶200, vol/vol). The new cultures were subsequently grown at 37°C until OD_600_ of 0.5 was reached in order to be induced with a final concentration of 0.01 mM isopropyl-1-thio-β-D-galactopyranoside (Fermentas, Thermo Fisher, Waltham, MA, USA) and 10 mM glucose following constant shaking overnight at 4°C. Cells were collected by centrifugation at 5400×g at 4°C for 15 min and resuspended in 2% of the original volume with 100 mM Tris/HCl, 500 mM NaCl, pH 8.0. The bacteria were sonicated on ice until complete lysis in four intervals of 30 seconds and lysates were clarified by centrifugation at 20,000×g for 30 min at 4°C. The recombinant soluble protein was first affinity purified via its N-terminal His-tag using Ni-NTA beads (Macherey and Nagel, Düren, Germany) and afterwards via its C-terminal Strep-tag using StrepTactin beads (IBA, Göttingen, Germany) according to the manufacturer’s instructions. Soluble proteins were loaded on Ni-NTA agarose and washed with 100 mM Tris/HCl, 20 mM imidazole, 500 mM NaCl, pH 8.0 buffer and eluted with 100 mM Tris/HCl, 250 mM imidazole, 500 mM NaCl, pH 8.0. The eluate was loaded on StrepTactin beads and washed with 100 mM Tris/HCl; 500 mM NaCl; pH 8.0 and eluted with 100 mM Tris/HCl, 2.5 mM desthiobiotin; 500 mM NaCl; 1 mM EDTA. The eluted recombinant proteins were dialysed using 10 kDa MWCO membranes against 20 mM NaHCO_3_, 500 mM NaCl, pH 8.7. One litre of LB media with transformed *E. coli* Rosetta (DE3) pLacI lead to approx. 0.5 to 2 mg pure fusion protein.

### CaCO_3_ Precipitation Assay

The method was performed as previously described [8], [45] with slight modifications. In brief, 250 µL of 20 mM NaHCO_3_, 500 mM NaCl at pH 8.7 with or without protein were mixed under constant stirring with 250 µL of 20 mM CaCl_2_, 500 mM NaCl. MBP-Perlucin or MBP dialyzed in 20 mM NaHCO_3_, 500 mM NaCl, pH 8.7 was added to the NaHCO_3_ solution before CaCl_2_ was added. As a control we used BSA or setups without any additives. The pH value was measured every 5 seconds for at least one hour using a thin Ross Micro pH electrode (Thermo Fischer, Waltham, USA) via pH meter (Portamess pH 910, Knick, Berlin, Germany) connected to a computer. After each measurement the pH-electrode was washed for 1 hour with HCl/Pepsin (AppliChem, Darmstadt, Germany). Afterwards, the pH-electrode was regenerated using 3 M KCl and then calibrated. For each splice variant three independent measurements were performed and both the average and the standard deviation were calculated. To address the concentration dependency only one measurement was performed.

### 2D Electrophoresis

Isoelectric focusing (IEF) was carried out according to the manufacturer’s instructions (BioRad, München, Germany) using immobilized pH gradient (IPG) strips (7 cm; BioRad) with a linear pH gradient of 3–10. For IEF native Perlucin purified from *H. laevigata* shell was acetone precipitated by adding 4 times the sample volume of ice-cold acetone and incubated overnight at −20°C. Precipitated Perlucin was centrifuged a 20,000×g at 4°C for 20 minutes and dissolved in 125 µL 8 M urea, 2% CHAPS, 50 mM dithiothreitol, 0.2% Bio-Lyte ampholytes (BioRad, Hercules, CA, USA). Following IEF (50 µA for 12 h, 250 V for 15 min, 250–4000 V for 2 h, 4000–20.000 V for 5 h) the IPG strip was equilibrated with SDS-PAGE buffer (6 M Urea, 2% SDS (w/v), 0,05 M Tris/HCl pH 8,7, 20% glycerol (v/v)) and placed onto a 12% SDS-polyacrylamide gel. SDS-PAGE was performed with 15 mA/gel. The 2D gel was stained with Coomassie Brilliant Blue.

### Western Blot Analysis

For Western blot SDS-PAGE (MiniProtean III; BioRad, Hercules, CA, USA) was performed and separated proteins were transferred to a polyvinylidene difluoride (PVDF) membrane (Millipore, Merck KG, Darmstadt, Germany). The membrane was blocked with 5% BSA in Tris buffered saline (TBS), 0.15% Tween 20™ for one hour. Immunodetection was carried out with a polyclonal rabbit anti Strep-tag antibody (1∶1000, IBA, Göttingen, Germany) in blocking buffer overnight. After washing four times with TBS, 0.15% Tween20™ a secondary donkey anti rabbit horseradish peroxidase conjugated antibody was applied (1∶20,000, Dianova, Hamburg, Germany) for two hours. After washing four times with TBS, 0.15% Tween20™ the membrane was developed with an enhanced chemoluminescence system (Amersham, GE Healthcare, Chalfont St Giles in Buckinghamshire, UK) and then exposed to X-ray film (Amersham).

### MALDI-ToF MS

Following 2D electrophoresis, Coomassie Brilliant Blue-stained protein spots were excised from gels and processed using Sigma’s Trypsin Profile IGD Kit as recommended by the manufacturer (Sigma, Deisenhofen, Germany). The extracted peptides were further purified by C_18_-ZipTip (Millipore, Billerica, MA, USA) and then premixed with a saturated matrix solution of α-cyano-4-hydroxycinnamic acid in 60% acetonitrile with 0.1% formic acid for spotting onto target plate. MALDI-ToF MS analysis was performed on an Autoflex III mass spectrometer in reflectron positive ion mode (Bruker Daltonics, Bremen, Germany). For each spectrum a minimum of 500 shots were accumulated in a mass range of 600–3,500 Da. For calibration a standard peptide mixture procured from Bruker Daltonics (Bremen, Germany) was spotted next to each sample and used as external control. After identifying Perlucin-derived peptides via a search against the SWISS-PROT database, an internal recalibration was performed using these peptides to reach highest mass accuracy.

### Liquid Chromatography Electrospray Ionisation Mass Spectrometry

For analysis peptides eluted from acrylamide gels (see above) were separated using an Ultimate 3000 RSLCnano system (Dionex-LC Packings, Idstein, Germany). Samples were loaded onto a trapping column (Acclaim PepMap Nanotrap, 75 µm×20 mm) by the loading pump of the system operating at 5 µL/min, and 0.1% trifluoroacetic acid in water was used as mobile phase. After 6 min, the valve was switched and the sample was eluted onto the analytical separation column (Acclaim PepMap RSLC, 75 µm×150 mm), using a flow rate of 300 nL/min. The mobile phases used were H_2_O/0.1% formic acid (v/v) for buffer A and 80% ACN/0.1% formic acid (v/v) for buffer B. Peptides were resolved by gradient elution using a gradient of 2−55% buffer B over 30 min, followed by a gradient of 50−90% buffer B over 2 min. After 5 min at 90% B the gradient returned to 5% buffer B in preparation for the next run. Column effluent was monitored using a 3 nL UV flow cell (214 nm). Mass spectrometric analysis was done via ESI-MS/MS using a LTQ-Orbitrap Velos mass spectrometer (Thermo Fisher, Bremen, Germany) equipped with a nano-electrospray ion source. The mass spectrometer was operated in the data dependent mode. Survey MS scans were acquired in the orbitrap with the resolution set to 30.000. Up to 15 most intense ions per scan were fragmented and analysed in the linear ion trap. The raw files were processed using Proteome Discoverer software version 1.2 (Thermo). The peak list files were searched against a homemade Perlucin database using the SEQUEST search engine. The initial parent and fragment ion maximum mass deviation were set to 8 ppm and 0.8 Da, respectively. The search included variable modifications of oxidation of methionine and carbamidomethylation of cysteine. The false discovery rate was set to 0.01.

### Determination of the Hydrodynamic Diameter

The hydrodynamic diameter of the three different splice variants in solution was determined by dynamic light scattering at a scattering angle of 90° using a L.i.SA light scattering machine (Fraunhofer IFAM, Bremen, Germany) [46]. Every splice variant was measured once after one month and again after two month in 20 mM NaHCO_3_, 500 mM NaCl, pH 8.7 at a concentration of 100 µg/mL, respectively.

The equations for the calculation of the theoretical hydrodynamic radii of the proteins were described elsewhere [47], [48]. In brief, for native proteins: ln(rh) = 0.29 ln (number of residues) +1.56, for denatured proteins: ln(rh) = 0.57 ln (number of residues) +0.79 were applied.

## Results

### Perlucin Splice Variants

**Figure 1:**
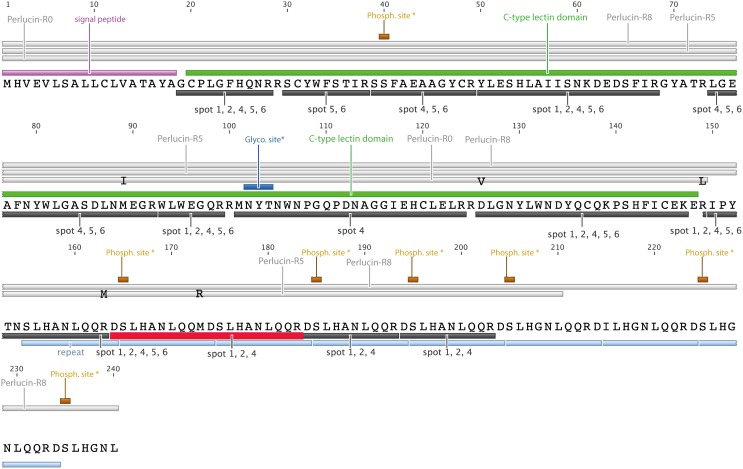
Amino acid sequences of Perlucin from *H. laevigata*. Perlucin splice variants (Perlucin-R0, Perlucin-R5, Perlucin-R8) are indicated as grey bars above the sequence. The following characteristics of the proteins are marked: Amino acid exchanges in Perlucin-R0 (M89I, V129D, R149L), signal peptide, C-type lectin domain [60], repeat units (light blue bars). Peptides identified from 2D electrophoresis spots (Figure 3) by MALDI-ToF MS (black bars) or ESI-MS (red bar). *Predicted glycosylation and phosphorylation (NetPhos [50]) sites.

Based on the amino acid sequence of purified Perlucin degenerate primers were designed and used in reverse transcriptase PCR (RT-PCR) with cDNA derived from mRNA of the mantle epithelia of *H. laevigata*. Starting from the resulting product of 330 bp coding for a Perlucin fragment 5′ and 3′ RACE experiments were performed to find the 5′and 3′ends of the mRNA. We obtained several clones coding for at least three different variants of Perlucin (Figure 1), named Perlucin-R0 (562 bp, 149 aa, [GenBank: FN674442.1]), Perlucin-R5 (734 bp, 210 aa, [GenBank. FN674444.1]) and Perlucin-R8 (815 bp, 240 aa, [GenBank: FN674445.1]), according to the numbers of sequence repeats at the C-termini of the encoded proteins. Whereas the 3′ends of cDNAs encoding for Perlucin-R5 and Perlucin-R8 are identical, they are different in Perlucin-R0 mRNA. This could be confirmed by the sequencing of different clones derived from different cDNA preparations. All these variants contain the same characteristic signal peptide leader sequence, predicted with high score by SignalIP [49].

The nucleotide sequences coding for the N-terminal 130 amino acids of the C-type lectin domain were almost identical in these variants (Figure 1). Only at 3 positions were sequence deviations leading to three amino acids exchanges (M89I, V129D, R149L) where found in several Perlucin-R0 cDNA clones, suggesting the existence of at least one additional allele. Consistent with this finding, additional Perlucin-R0 cDNA clones (562 bp, 149 aa [GenBank: FN674443.1]) were later found, encoding the same amino acids at positions 89 and 126 as Perlucin-R5 and Perlucin-R8.

Perlucin-R0 basically consists of the C-type lectin domain, whereas Perlucin-R5 and Perlucin-R8 have 5 or 8 repeats of the 10 amino acids sequence SLHA(G)NLQQR(M)D, respectively. These repeats are highly conserved within the cDNA of Perlucin splice variants. According to the NetPhos 2.0 Server [50], the serine residues of the repeats are potential phosphorylation sites with a high score (0.95) unless the previous repeat contains a methionine instead of arginine at the eighth position (Figure 1). This results in six potential phosphorylation sites in the repeat region of Perlucin-R8.

### Phylogeny

**Figure 2:**
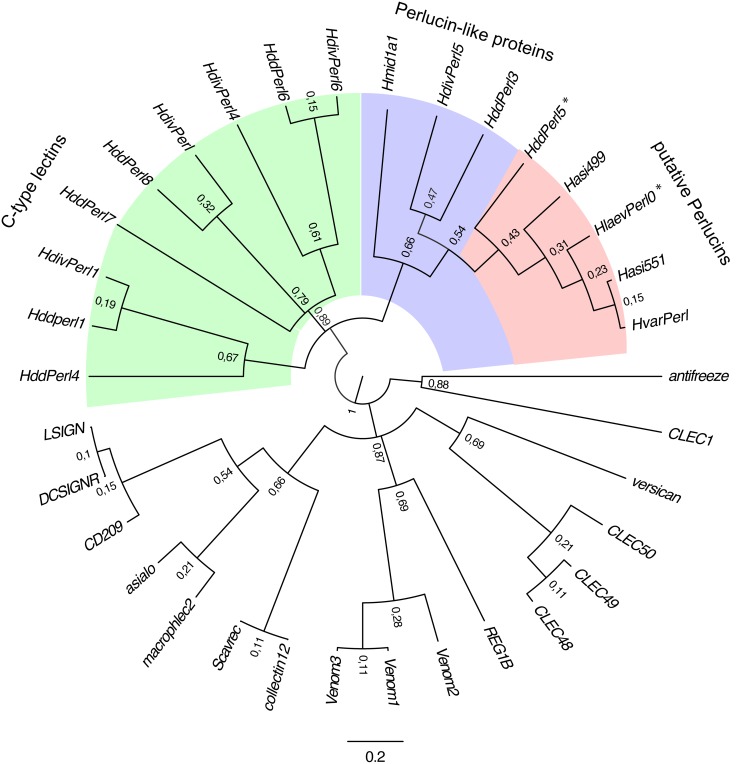
Phylogenetic tree of Perlucin and related proteins. DNA Fragments coding for proteins related to Perlucin were selected as described in the Methods section. The phylogenetic tree was generated using Geneious [36] plugin MrBayes [43] with HKY85 substitution model [41] and 4 invariant gamma rate categories, (HKY85 G+I) and type II antifreeze glycoprotein from the Atlantic herring (*Clupea harengus*) as out-group. Highlighted in pink are the putative Perlucins, in blue, Perlucin–like proteins and in green, proteins with C-type lectin domain from *Haliotis* spec. Asialo = asialoglycoprotein receptor H1, *Homo sapiens*; CD209 = DC-SIGN, *Homo sapiens*; collectin12 = collectin sub-family member 12, *Homo sapiens*; DCSIGNR = sDC-SIGN1A type III, *Homo sapiens*; LSIGN, *Homo sapiens*; macrophlec2 = macrophage lectin 2, *Homo sapiens*; REG1B = regenerating islet-derived 1 beta, *Homo sapiens*; Scavrec = Scavenger receptor, *Homo sapiens*; versican = chondroitin sulfate proteoglycan 2, *Homo sapiens*; HlaevPerl0* = Perlucin R0, *Haliotis laevigata*; HvarPerl = putative Perlucin, *Haliotis varia*; Hasi551 = *Haliotis asinina*; Hasi499 = *Haliotis asinina*; Hddperl1 = putative Perlucins 1, *Haliotis discus discus*; HddPerl3 = putative Perlucin 3, *Haliotis discus discus*; HddPerl4 = putative Perlucin 4, *Haliotis discus discus*; HddPerl5* = putative Perlucin 5, *Haliotis discus discus*; HddPerl6 = putative Perlucin 6, *Haliotis discus discus*; HddPerl7 = putative Perlucin 7, *Haliotis discus discus*; HddPerl8 = putative Perlucin 8, *Haliotis discus discus*; HdivPerl = putative Perlucin, *Haliotis diversicolor*; HdivPerl1 = putative Perlucin 1, *Haliotis diversicolor*; HdivPerl4 = putative Perlucin 4, *Haliotis diversicolor*, HdivPerl5 = putative Perlucin 5, *Haliotis diversicolor*; HdivPerl6 = putative Perlucin 6, *Haliotis diversicolor*; Hmid1a1 = putative Perlucin, *Haliotis midae*; Venom1 = Venome gland lectin 1, *Bungarus fasciatus;* Venom2 = Venome gland lectin 2, *Bungarus fasciatus*; Venom3 = Venome gland lectin 3, *Bungarus fasciatus*.

In nucleotide databases several genes of *Haliotis* species have been assigned as (potential) Perlucins. Since the group is quite heterogeneous, we tested their phylogenetic relationships to Perlucin, focussing on the C-type lectin domains of gene products from *Haliotis* spec. assigned as Perlucin in the databases (Figure 2) in comparison to C-type lectins from other animal species. The resulting tree is divided into two main groups. One group represents the C-type lectins from *Haliotis* spec., whereas the other group comprises C-type lectins from vertebrate and other invertebrate species. The *Haliotis* spec. group is divided into several branches. Interestingly, genes from *H. discus discus* are distributed over different branches. Only one of these (HddPerl5), which has experimentally been confirmed as Perlucin [14], is closely related to *H. laevigata* Perlucin. This cluster most likely represents real Perlucins (Figure 2) comprising further genes from *H. varia* and *H. asinina*.

Another set of genes, including HddPerl3, although less related to the Perlucin cluster, is clearly separated from the majority of other genes encoding C-type lectins from *H. discus discus* and *H. diversicolor*. These “Perlucin-like” genes might encode for active Perlucins, due to their similarity, but this needs to be verified experimentally.

### C-terminal Repeats in Native Perlucin

**Figure 3:**
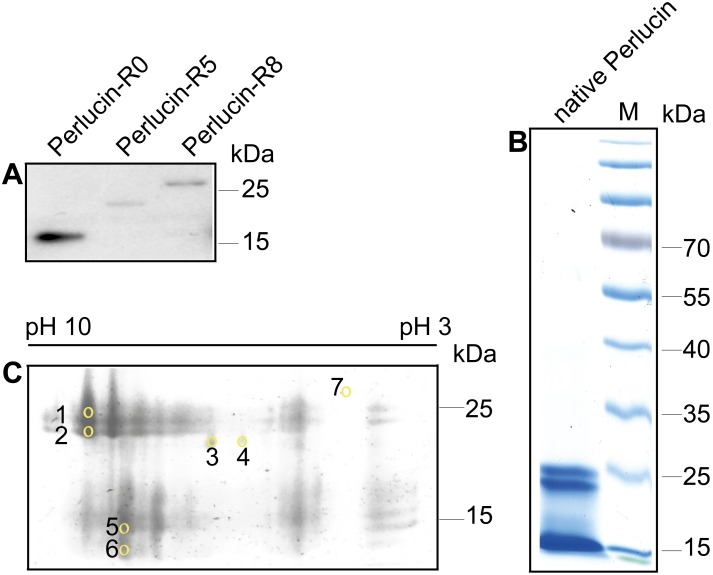
Electrophoretic analysis of recombinant and native Perlucin preparations. A) Western blot of cell culture supernatants derived from COS-7 cells ectopically over expressing the indicated Strep-tagged recombinant Perlucins, which were detected using a polyclonal anti-Strep-tag antibody as described in the Methods section. B) SDS-PAGE of native Perlucin purified from abalone shell of *H. laevigata*, stained with Coomassie brilliant blue showed one distinct band at approx. 25****kDa, one at 20****kDa, and one at approx. 15****kDa. C) 2D electrophoresis of native Perlucin purified from abalone shell of *H. laevigata*, stained with Coomassie Brilliant Blue. The indicated spots were cut out and analysed by MALDI-ToF MS as described in the Methods section. Spot 7 was used as control.

The discovery of Perlucin mRNA splice variants encoding for proteins with different C-termini raised the important questions, of (I) whether these occur in native protein from nacre and (II) whether they might be the reason for the heterogeneity of purified Perlucin. If expressed in COS-7 cells, C-terminally Strep-tagged recombinant Perlucin variants migrate in SDS-PAGE at 15 kDa (Perlucin-R0), 20 kDa (Perlucin-R5) and 25 kDa (Perlucin-R8) (Figure 3A). The electrophoretic migration behaviour of the recombinant proteins correlates quite well with those observed for Perlucin preparations from the shell of *H. laevigata* using a standardised protocol [13] (Figure 3B). Native Perlucin appeared as several spots on 2D electrophoresis in a broad pH range from approximately 5.0 to 9.5 and apparent molecular masses from 27 kDa to 10 kDa (Figure 3C) representing the three prominent distinct bands detectable in SDS-PAGE at 25 kDa, slightly below 25 kDa and 15 kDa (Figure 3B). Seven spots from the 2D electrophoresis (Figure 3C) were selected for MALDI-ToF MS analysis and digested with trypsin: Spots 1 and 2 in the range of 25 kDa, spots 3 and 4 at approximately 20 kDa, spot 5 at 15 kDa, spot 6 below 15 kDa.

Most of the peptide masses expected from the published Perlucin sequence [12] were identified in all samples (except spot 3) demonstrating that these spots represent Perlucin (Table 2). The peptide containing the single putative *N*-glycosylation site was detected only in spot 4 (Figure 1). As, depending on their sequence, tryptic peptides show different ionization properties in matrix assisted laser desorption ionization (MALDI) or electrospray ionization (ESI), we reanalysed all spots by LC-ESI-MS so as to not miss any sequence information. In fact through this method we found an additional peptide DSLHANLQQMDSLHANLQQR (Figure 1) including the repeat sequence found in the C-termini of the splice variants Perlucin-R5 and Perlucin-R8, but not Perlucin-R0, in spot 1 and 2, in low intensity in 4, but not in spot 5 and 6. These findings clearly demonstrate the presence of Perlucin variants in the organic material of nacre.

**Table 2:** MALDI-ToF MS results.

Spot No	Perlucin identified	Mascot sore	Sequence coverage	#Peptides
1	yes	81	52%	6
2	yes	79	52%	6
3	no	-	-	-
4	yes	79	73%	8
5	yes	81	72%	9
6	yes	92	72%	9
7	no			

Trypsin digested peptides extracted from 2D electrophoresis were analysed by MALDI-ToF MS. Masses of peptides were used to search an in house SwissProt database in all entries mode. Sequences of peptides are shown in Figure 1.

### Recombinant Protein Expression and Purification

**Figure 4:**
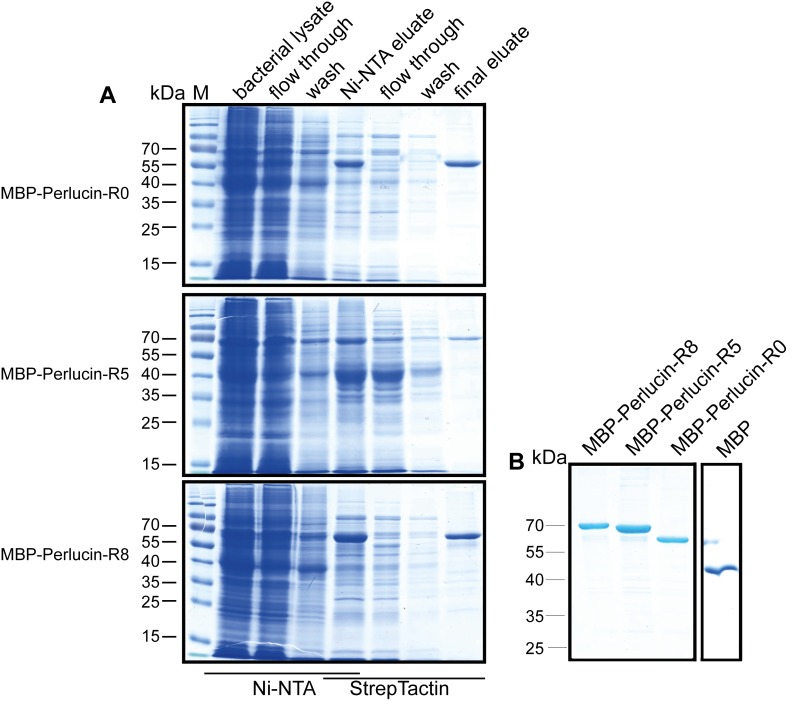
Purification of Perlucin splice variants fused with MBP. Recombinant proteins were expressed in *E. coli* Rosetta (DE3) pLacI and purified via N-terminal His-tag and C-terminal Strep-tag of MBP-Perlucin-R0 (60****kDa), MBP-Perlucin-R5 (68****kDa) and MBP-Perlucin-R8 (72****kDa) and MBP (42****kDa) as described in the Methods section. (A) Samples representing the purification steps. (B) Purified proteins after dialysis.

For further characterization the splice variants of Perlucin were expressed in *E. coli* as fusion proteins, each with His-tagged MBP and Strep-tag at the N- and C-termini, respectively. This allowed tandem tag affinity purification first via the His-tag and then via the Strep-tag leading to a high purity of the proteins, as demonstrated by SDS-PAGE. As a control MBP without Perlucin was prepared accordingly (Figure 4).

### Calcium Carbonate Precipitation Assay

**Figure 5:**
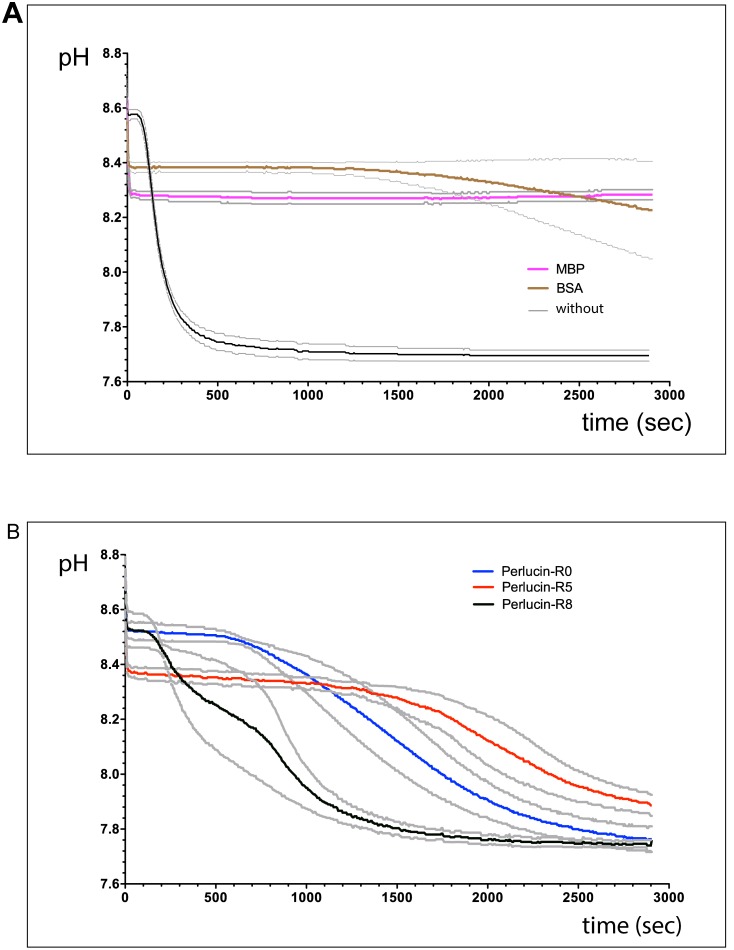
Enhancement of CaCO_3_ precipitation by MBP-Perlucin. 250** µ**L of 20****mM NaHCO_3_, 500****mM NaCl, pH 8.7 containing 100** µ**g/mL of the indicated protein were mixed with 250** µ**L 20****mM CaCl_2_, 500****mM NaCl, pH 8.7 and the pH was recorded to monitor CaCO_3_ precipitation as described in the Methods section. The average pH values of three independent measurements (coloured) with standard deviations (grey) are shown. A) Control reactions with MBP (pink), BSA (brown), or without protein (black). B) MBP-Perlucin-R0 (blue), MBP-Perlucin-R5 (red), MBP-Perlucin-R8 (black).

Critical questions were (I) whether the recombinant variants of Perlucin modulate the precipitation of CaCO_3_ and (II) whether the absence or presence of the C-terminal repeats and their number has an influence on this function of Perlucin. To investigate this, we used a CaCO_3_ precipitation assay with the MBP fusion proteins. A similar assay [13] had been used to establish that native Perlucin accelerates the precipitation of CaCO_3_. Furthermore, this assay has been also performed with a recombinant MBP-Perlucin fusion protein [14] derived from *H. discus discus*. As expected for functional Perlucin, the recombinant MBP-Perlucin fusion proteins lead to an accelerated precipitation of CaCO_3_ compared to MBP alone. When tested with 100 µg/mL freshly prepared MBP-Perlucin splice variants, significant differences on the precipitation behaviour of CaCO_3_ were observed with an increasing effect in the order from MBP-Perlucin-R0 to MBP-Perlucin-R5 to MBP-Perlucin-R8 (Figure 5B) compared with 100 µg/mL BSA or MBP alone (Figure 5A).

**Figure 6:**
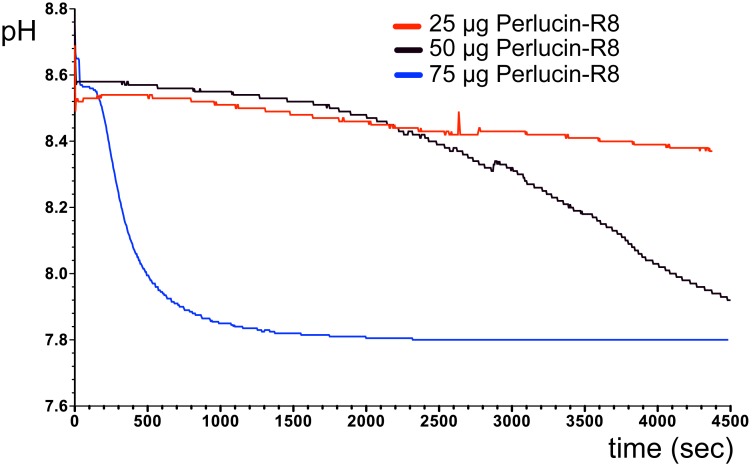
Perlucin concentration dependency of CaCO_3_ precipitation. In order to obtain equal protein concentrations in all assays, the following mixtures of MBP and MBP-Perlucin-R8 were used in CaCO_3_ precipitation assays as described in the Methods section: 50** µ**g MBP with 25** µ**g MBP-Perlucin-R8 (red); 25** µ**g MBP with 50** µ**g MBP-Perlucin-R8 (black); 75** µ**g MBP-Perlucin-R8 (blue).

In a next step, we addressed whether this accelerating effect on CaCO_3_ precipitation depends on Perlucin concentrations. Therefore, we set up assays in which we combined different amounts of MBP-Perlucin-R8 with MBP added to a total of 75 µg protein. Under these conditions, 25 µg MBP-Perlucin-R8 barely accelerated the precipitation of CaCO_3_, whereas in the presence of 50 µg MBP-Perlucin-R8 precipitation started after approx. 2,500 seconds and with 75 µg MBP-Perlucin-R8 accelerated precipitation of CaCO_3_ was even stronger after approx. 500 seconds with a pH shift from pH 8.6 to pH 7.9 (Figure 6). For MBP-Perlucin-R0 a similar concentration dependency on CaCO_3_ precipitation was observed.

### Determination of the Hydrodynamic Diameter

The stronger accelerating effect of MBP-Perlucin-R5 and MBP-Perlucin-R8 on CaCO_3_ precipitation could be due to the formation of higher order Perlucin complexes induced by the C-terminal peptide repeats. This hypothesis was supported by the observations that storing Perlucin preparations for longer time periods leads to faster precipitation of CaCO_3_. To investigate the formation of Perlucin oligomers, we determined the hydrodynamic diameters of the different Perlucin variants stored for one or two months in 20 mM NaHCO_3_, 500 mM NaCl, pH 8.7 using the same protein concentrations. For comparison we used MBP, which shows almost no aggregation (hydrodynamic diameter below 10 nm). In contrast, after one month the hydrodynamic diameters for all Perlucin variant fusion proteins were at least 10 times larger and after 2 months had increased further to almost 100 times greater than the theoretically calculated diameter for MBP-Perlucin-R5 and MBP-Perlucin-R8 containing 5 or 8 repeats, respectively, which showed a bimodal particle size distribution and formed aggregates of up to 750 nm diameter (see Table 3).

**Table 3:** Hydrodynamic diameter of recombinant Perlucin splice variants.

	1 month	2 month	“native” theoretical rh	“denatured” theoretical rh
CaCO_3_	<10 nm			
MBP	<10 nm	<10 nm	2,7 nm	6,8 nm
MBP-Perlucin-R0	<10 nm	45 nm	3,1 nm	8,8 nm
MBP-Perlucin-R5	130 nm	42/860 nm	3,2 nm	9,3 nm
MBP-Perlucin-R8	91 nm	49/760 nm	3,2 nm	9,4 nm

Hydrodynamic diameters were determined as described in the Methods section after storing in 20****mM NaHCO_3_ 500****mM NaCl pH 8.7 for one or two months. Theoretical hydrodynamic diameters were calculated as described [47], [48].

## Discussion

Analysis of purified native Perlucin from the shell of *H. laevigata* demonstrated a high degree of heterogeneity [12]. In this study we show that at least in part this derives from alternative splicing. Three new Perlucin splice variants were identified in cDNA from *H. laevigata*, differing mainly in the number (zero, five or eight) of 10 mer repeats (DSLHANLQQR) at the C-terminus. Sequencing of purified native Perlucin has already provided evidence for two repeats [12]. However, since the repeats consist of almost the same amino acid sequences, the variable number of these repeats cannot be determined by protein sequencing.

The mass spectrometry data of purified native Perlucin digested with trypsin clearly showed that these variants are present in the shell of *H. laevigata*, since a NH_2_-DSLHANLQQMDSLHANLQQR-COOH peptide was identified in protein spots with apparent molecular weights of 25 kDa and 20 kDa, but not in the spots 5 and 6 appearing at around 15 kDa (Figure 3). Interestingly, this peptide does not match the sequence by Mann et al [12], but is consistent with the cDNA sequences encoding Perlucin-R5 and Perlucin-R8 (Figure 1). Current database information is insufficient to identify such repeats in Perlucin from other species. However, it is quite possible that Perlucin variants with similar repeats will be identified in other species of the genus *Haliotis* using a similar strategy.

Several genes or proteins have been assigned as Perlucin or Perlucin-like in DNA and protein databases. To address this remarkable diversity we performed a phylogenetic analysis (Figure 2). The two proteins for which Perlucin activity has been reported from *H. laevigata* (HlaevPerl0) and from *H. discus discus* (HddPerl5) [12]–[14], [32] are closely related to the putative proteins from *H. asinina* (Hasi551, Hasi499) and *H. varia* (HvarPerl). This high degree of similarity suggests that they are Perlucin orthologs and therefore their assignments as Perlucins are likely to be justified. Several other genes from *H. discus discus* and *H. diversicolor* have also been assigned as Perlucins. Only three of these genes share the main branch with Perlucins and are therefore considered to be Perlucin-like proteins, whereas the other genes are only distantly related to Perlucin and are clustered in a separate branch, thus it is unlikely that they have Perlucin-like properties, in particular the promotion of CaCO_3_ crystallisation, and probably represent other C-type lectins. Obviously, clarification of the experimental evidence of all proteins is necessary in order to determine which of these can be classified as Perlucin.

Recently, Perlucin-like proteins have been found in the transcriptome of *Mytilus edulis* [51] and in the genome study of *Pinctada fucata* [52]. Although there is as yet no experimental evidence that these genes are involved in shell formation in bivalves [52], Perlucin and Perlucin-like proteins may be conserved proteins in bivalve and gastropods, derived from a common ancestor.

In addition to alternative splicing, posttranslational modifications clearly lead to heterogeneity. One motif for *N*-glycosylation has been identified in Perlucin [12]. Our MALDI-ToF MS data demonstrate that the non-glycosylated protein also occurs, since the corresponding non-glycosylated peptide was identified in the peptide map of spot 4 (20 kDa, Figure 1). Obviously, this does not exclude the existence of glycosylated forms of Perlucin exist. Since the glycan structures of *Haliotis* are unknown, the molecular masses of the corresponding glycopeptides cannot be predicted and no evidence for the non-glycosylated peptide was found in any of the other spots. Another important posttranslational modification might be phosphorylation, which for example positively influences calcium binding by Orchestin, a soluble matrix protein of the crustacean calcium storage structures [53]. Purified native Perlucin consists of several species of different isoelectric points (Figure 3B) consistent with patterns observed for phosphorylation and supported by several predicted serine phosphorylation sites.

One important functional parameter for Perlucin is its ability to accelerate the precipitation of CaCO_3_ from a saturated solution [13] [32]. Apparently this important function is modulated by the repeats, since the precipitation velocity increases with their number (Figure 5) and *H. laevigata* Perlucin-R8 led to the most pronounced and fastest CaCO_3_ precipitation in a concentration dependent manner (Figure 5). This is in good agreement with CaCO_3_ precipitation induced by native Perlucin from *H. laevigata* [13]. Perlucin from *H. discus discus* (no repeats) fused to MBP accelerated the precipitation of CaCO_3_ [14], at a rate similar to *H. laevigata* Perlucin-R0 (Figure 6), whereas recombinant *H. laevigata* Perlucin (two repeats) fused to GFP did not accelerate the precipitation of CaCO_3_ compared to control protein Concanavalin A or even prevented the precipitation, if added as a CaCl_2_ solution [34].

In contrast to Perlucin, other proteins appear to stabilize the supersaturated CaCO_3_ solutions, as shown for MBP and BSA (Figure 5A). In this context it is important to note that crude soluble matrix proteins from the shells of *H. laevigata* [45] or *Crassostrea virginica* [8] inhibit crystals growth of CaCO_3_. Thus, it is tempting to speculate that the balance between crystallisation-inhibiting matrix proteins and Perlucin controls the crystal growth in nacre. Our observation that the splice variants promote CaCO_3_ precipitation at different rates supports the idea that the repeats play a role in the fine tuning of aragonite formation. Along this line, it is interesting to note that the number of repeats influences the rate of higher order Perlucin complex formation (Table 3).

Other C-type lectins oligomerise via C-terminal extensions, such as the mannose-binding protein A with its collagen like C-terminus [54], collectins [55] or tetranectins [56]. Although the C-terminal repeats do not resemble collagen, they might act in a similar way to oligomerise Perlucin leading to the observed aggregates (Table 3). Recently, Evans *et al.* proposed [35] that the repeats described here might represent an intrinsically disordered region. This would explain the protein aggregates of almost 1 µm that we observed for the splice variants R5 and R8. Similarly, a function of intrinsically disordered regions has been proposed for protein r-n16.3 from pearl oyster, which oligomerises in a wide pH range and the radii of these oligomers increase in the presence of Ca^2+^ [57] and the abalone nacre protein AP7 [58]. Furthermore, Parham *et al*. [59] demonstrated that the N-terminal repeats of the yeast protein Sup35p stabilise protein aggregates similar to the human prion protein PrP.

Although the dynamic light scattering results have to be considered as “preliminary” and more extensive studies concerning this phenomenon have to be addressed, they provide evidence for a very interesting functional aspect of the repeats: Perlucin-R5 and Perlucin-R8 may oligomerise in the same way and this oligomerisation could lead to the large aggregates we found. And these large aggregates could in turn lead to a faster precipitation of CaCO_3_, fine-tuned depending on of the length of the repeats.

## Conclusions

Alternative splicing of *H. laevigata* Perlucin leads to variants with different number of C-terminal 10 mer peptide repeats. With increasing number of repeats Perlucin forms aggregates with a hydrodynamic radius of up to almost 1 µm. Interestingly, the number of repeats also correlates with the promotion of CaCO_3_ precipitation, a hallmark of Perlucin.

It has been hypothesised that the accumulation of secreted Perlucin leads to aggregates serving as nucleation seeds, thereby accelerating the precipitation of CaCO_3_. In agreement with this hypothesis, native *H. laevigata* Perlucin and Perlucin-GFP fusion constructs are incorporated in CaCO_3_ crystals [32], [34]. Crystal growth could be further influenced by other proteins of the organic matrix acting as promoters or inhibitors of this process. Along this line, alteration of expression rates and secretion levels of the Perlucin splice variants reported here, with their altered aggregation behaviour, appear ideal for modulating rate and composition of aragonite platelets and thus fine tuning nacre formation.

## Supporting Information

File S1 **cDNA alignment used for phylogenetic analysis.** As described in the Methods section the cDNA encoding the C-type lectin sequences were aligned with T-coffee [61] using the AS sequences and reverse translation were done with Revtrans 2.0 [40]. This txt-file is in fasta format.
(TXT)
